# Signal enhancement techniques for chromatography detection systems

**DOI:** 10.1155/S146392469700014X

**Published:** 1997

**Authors:** K. D. Jackson, S. J. Walton, D. Campbell

**Affiliations:** Thomas Swan & Co. Ltd Crookhall Co. Durham Consett UK

## Abstract

The Environmental Protection Act has created a growing need for the measurement and assessment of trace emissions to the environment. This encompasses three main areas of ground, water and the atmosphere. The need to achieve lower emissions has placed a large burden on analytical techniques, particularly in the areas of trace analysis to ppb and ppt levels. Chromatographic techniques are widely used for assessment and measurement of emissions in all three areas. Enhanced detectors using mass spectrometry principles are available to lower detection limits, but these are expensive. Standard chromatography detectors can be used for trace analysis, but this often leads to extensive sample preparation stages to achieve low detection limits. This paper describes the techniques developed by Thomas Swan & Company to introduce a cost effective way of lowering detection limits. The approach taken meets both BATNEEC and BPEO constraints.


Journal of Automatic Chemistry, Vol. 19, No. 5 (September-October 1997) pp. 145-152

### Signal enhancement techniques for chromatography detection systems

**K. D. Jackson, S. J. Walton and D. Campbell**
*Thomas Swan & Co. Ltd, Crookhall, Consett, Co. Durham, UK*

*The Environmental Protection Act has created a growing need for the measurement and assessment of trace emissions to the environment. This encompasses three main areas of ground, water and the atmosphere. The need to achieve lower emissions has placed a large burden on analytical techniques, particularly in the areas of trace analysis to ppb and ppt levels. Chromatographic techniques are widely used for assessment and measurement of emissions in all three areas. Enhanced detectors using mass spectrometry principles are available to lower detection limits, but these are expensive. Standard chromatography detectors can be used for trace analysis, but this often leads to extensive sample preparation stages to achieve low detection limits. This paper describes the techniques developed by Thomas Swan & Company to introduce a cost effective way of lowering detection limits. The approach taken meets both BATNEEC and BPEO constraints.*

### Background

As a manufacturer of a wide range of speciality chemicals, Thomas Swan & Co. have always faced the issue of the safe containment and disposal of the chemical by-products from its processes to meet ethical and legislative requirements. The company makes strong efforts to minimize its impact on the local environment, as evidenced by early compliance and registration with HMIP under the EPA90 Act and certification to BS7750—the company was one of the test sites for this standard and was one of the first to be awarded certification. The company has a relatively large research and development department that develops new products and processes and works on the overall systems and processes used in manufacturing, including BS5750 and BS7750 compliance and emission monitoring and control. Included under this research and development umbrella is an internal instrumentation activity set up to adapt and improve analytical equipment systems—especially their electrical and electronic performance. This department's work has included the redesign and adaptation of the electronics of a wide range of chromatography equipment. For example, the sensitivity obtained from an in-house modified Flame Ionisation Detector (FID) system on an old Philips PU4500 Gas Chromatograph is significantly better than the system as originally specified and delivered. Indeed, the performance is comparable to current state of the art systems.

Evolving out of this understanding of chromatographic processes and equipment have come the signal enhancement techniques discussed in this paper.

### Sensitivity improvement

Sensitivity is an issue in chromatography particularly in the fields of product quality, waste monitoring and emission control where legislative trends are to enforce the lowest possible levels of emission and highest possible standards of control. This quest for improved sensitivity has significant process and economic consequences.

The typical reaction to new demands is method development to make the sample performance fit the available equipment performance—solid phase extraction and supercritical fluid extraction being two examples—or the adoption of increasingly more sophisticated equipment concepts to everyday tasks—the increasing use of mass spectroscopy for example. Both approaches adding to the complexity, cost and time associated with analysis. Of course original equipment performance also evolves under this pressure for higher sensitivity, but seemingly somewhat slowly.

At Thomas Swan the approach has been to look at the main limitations of standard chromatography equipment in these new more sensitive applications.

Although, at first, process limitations—for example sample preparation, column performance and operational procedure—seem paramount, a major area of limitation is in the processing of the chromatographic signal. Improved signal to noise in the process and detection equipment and in the down-stream signal processing almost always leads to an improved level of sensitivity. The questions are how much improvement is available before other limitations impinge and how to achieve any improvement most cost effectively. Of course there will always be some problems that only the last resort of newer more specialized equipment can solve, but a surprising amount of progress can be achieved before this is necessary.

Early attempts at improving the detection and down-stream processing equipment focused mainly on understanding the sources of noise within the system and the redesign of the analogue electronics surrounding the chromatography sensors to counter them. Careful attention to design, component selection and circuit topography can all have significant impacts on performance and induced noise.

### Understanding noise

One of the first requirements to improving noise performance is to understand the sources of noise within the overall system. From the chemical point of view, noise can arise from a wide variety of sources including contamination, process interaction and changes in the

0142-0453/97 $12·00 © 1997 Taylor & Francis Ltd process environment. Careful attention to the detail of process design will minimize the chemical noise presented to the detector. At a system level, chromatography is also an inherently noisy process. Irregularities in the eluent pressure and flow rate, thermal effects, and column binding effects, all conspire to provide the detector with a noisy signal. Again attention to detail and equipment set-up can help minimize these effects. Once at the detector the detection process itself is generally inherently noisy. In gas chromatography the nominal flame ionization detector is extremely noisy, with carrier gas impurities and pressure variations, thermal effects, high voltage power supply noise and ionization noise. In liquid chromatography, UV detectors are subject to stray light, cell contamination, source fluctuation, source ageing effects and sensor shot noise. Once the sample constituents have been detected, and converted to an electronic signal, there is still the noise resulting from the amplification and signal processing circuits, including Johnson noise, shot noise, flicker noise and all of the various effects of electromagnetic radiation, power-supply noise and thermally induced currents to add to the mess.

To take just one simple example, the noise contribution from a resistor is defined by

$$E_t = (4kTRF)^{1/2}$$

where $k$ = Boltzmann's constant ($1{\cdot}38 \times 10^{-23}$)
$T$ = operating temperature in °K
$R$ = resistor value in Ohms
$F$ = signal bandwidth frequency.

For a nominal 1000 ohm resistor at 25°C with band limiting to 10 Hertz the noise contribution is a seemingly insignificant 12·8 nano-volts. However if this resistor is somewhere in the input or feedback circuits of our FID amplifier with 1,000,000,000,000 × amplification, the effect of this noise can be somewhat more significant! It is important to recognize that any noise appearing in a circuit is subject to the same amplification as the signal from that point on.

Figure 1 shows a generic model of a typical FID amplifier. The amplifier is configured as a current to voltage amplifier to try to minimize the effects of the various voltage noise components present in an amplifier at such a sensitive stage. This front end would normally be followed by several voltage gain stages to amplify the signal to the levels required by the downstream processing elements.

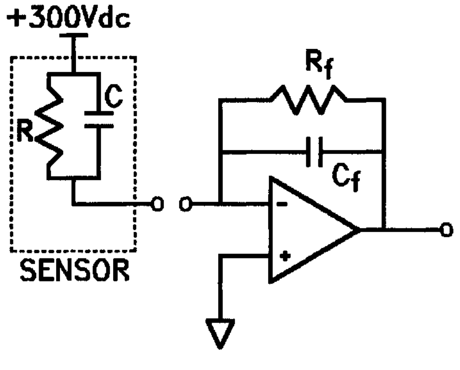

*Figure 1. A generic model of a current-to-voltage amplifier as used in a typical FID amplifier.*

In a current to voltage configuration, the major noise contributors are shot noise based on the bias current flowing in the amplifier feedback loop via resistor $R_f$ and the amplifier's current input noise.

Shot noise is defined by:

$$V_s = (2qL_bF_b)^{1/2}$$

where $q$ = electron charge, $1{\cdot}6 \times 10^{-19}$

$I_b$ = bias current, $\sim 2 \times 10^{-12}$

$F_b$ = frequency bandwidth.

In the example shown, shot noise can contribute approximately 8·0 mV of noise at the output of the amplifier or about 1% of the full scale range of the output. This is without considering the amplifier's internal input voltage noise and current noise contributions, other resistor noise, power supply ripple and feedthrough noise and the effect of any electromagnetic or electrostatic induced noise in the wiring of the amplifier. It is easy to see that seemingly 'insignificant' noise factors can accumulate into a major problem.

Attention to design topology, component selection, layout, power supplies and screening can all help to minimize noise contribution. However, the physical limitations of even the best of today's analogue electronic technology combine to constrain the overall performance that can be extracted from any given analogue system.

If a system is noisy, a logical approach is to apply some form of signal filtering to try to extract the signal from the noise. After improving the core circuit design of systems, Thomas Swan moved towards using analogue filters to help isolate the signal. It is interesting to note here that the authors were not alone in this approach. In many cases the standard 'industry' answer to the noise problem has been to apply an analogue filter to the signal. Most manufacturers, however, use the simplest of circuitry to just 'squash' the noise, while accepting the distortion of the signal that results.

Figure 2 shows a typical two pole Sallen-Key filter circuit. The circuit values are calculated for a corner frequency of 15 Hz. Note the large value components involved.

Figure 3 shows the frequency response of the Sallen-Key filter circuit in figure 2. Notice the slow roll off.

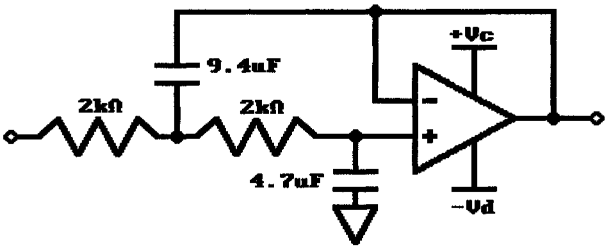

*Figure 2. Two pole Sallen-Key filter circuit.*

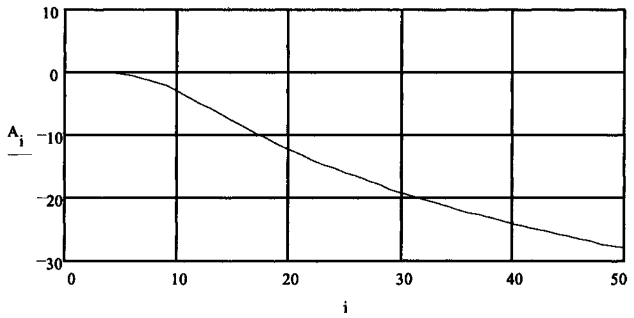

*Figure 3. Frequency response of filter circuit.*

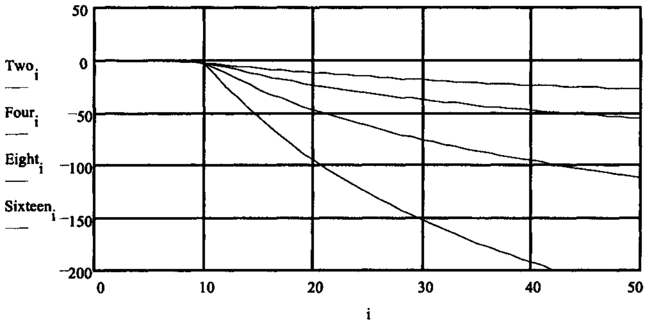

*Figure 4. Cascading effects.*

Analogue filtering, while simple in concept, rapidly falls foul of the compromises inherent in balancing the induced noise (and cost) added by additional complexity against the complexity needed to attain the filter performance required. To obtain improved performance from analogue filters, the filters are cascaded with each filter block contributing a factor of two to performance. However, even large cascaded filters still offer relatively low performance in the real world.

Figure 4 shows the impact on frequency response of cascading two, four, eight and 16 filter stages.

It is important to realize that in chromatography the ultimate goal is to measure peak height and peak area. Filtering that affects either peak height or peak shape can have a significant impact on the peak measurements.

Having tried and found analogue signal processing wanting, attention was turned to the possibilities of digital signal processing.

### The digital approach

Digital filtering and signal processing techniques are well established and a wide range of literature offers a host of different approaches to extracting a 'known' signal from noise. A point to note here is that in chromatography one is usually searching for an 'unknown' signal and that, in general, chromatography produces fairly complex signals.

Generically, digital signal processing offers significant advantages over its analogue equivalent. Digital processing is inherently noise non-additive—the number you start with is substantially maintained whatever the later

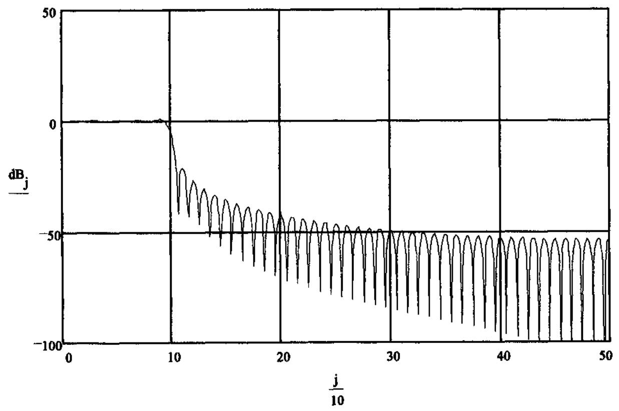

*Figure 5. Frequency response, moving average filter.*

processing—at least within the bounds of the effects of rounding errors. Compare this with analogue processing where each additional stage contributes additional noise to the signal. Also, complexity in the processing algorithm need not degrade the validity of the signal, and filter algorithms exist that offer significantly greater performance than is available in the analogue domain. The downside is that digital signal processing can be extremely computationally intensive, especially when faced with a signal with a large dynamic range and a complex signature. In addition the familiar computing expression 'garbage in, garbage out' is especially true leading to a continued requirement for excellent analogue design in the pre-digital and analogue-to-digital conversion stages. Even the best signal enhancement algorithms cannot retrieve a signal that is not there.

### Digital signal processing techniques

There are many different approaches to digital signal processing. All, however, are based on the sampling of the analogue signal on a regular basis via an analogue-to-digital converter. At the simplest level, the signal processing can be just 'bunching' where the signal is over-sampled by a factor of $N$ and $N$ samples are accumulated and averaged to produce the user's sample rate. This simple technique is the basis of many of the high resolution sigma-delta A/D converters used in today's chromatography data collection systems. Bunching reduces the noise by a factor of $\sqrt{N}$, i.e. 100 times over-sampling gives a factor of 10 (or a −20dB) reduction in noise.

Similar to bunching is the technique of using a moving average, where, instead of over-sampling and bunching the data, the data are collected at the required sample rate and stored in memory. The current sample value is calculated from an average of the last $N$ samples. Depending on the sample rate and the value of $N$, this can be a significant improvement on the bunching technique.

Figure 5 shows the frequency response calculated for a moving average filter based on a sample rate of 100 samples per second and an order $N = 101$. It can be seen that the initial roll-off is much faster than for a complex multi-stage cascaded analogue filter.

A more complex method of averaging uses the idea of weighting the various sample values accumulated into the average. An example of this technique is the Savitsky-Golay algorithm. In this algorithm the various samples accumulated are weighted according to their time position in the sequence. A wide variety of pre-calculated weights are available in published form, most of them based on a least-squares error function analysis between the algorithm and a target response.

With all of these simple averaging techniques, the main drawback is that compared to more efficient processing algorithms they are still slow to roll-off (typically ~10 dB/octave) and have a relatively low noise rejection performance (typically ~40 dB).

Many of the more efficient signal processing algorithms are based on the FIR (finite impulse response) digital filter. Superficially these fit into the weighted averaging class of algorithms. However, the performance available from the algorithms used to generate the weights is significantly better than with either moving average or Savitsky-Golay approaches. FIR algorithms are able to implement a wide range of filter profiles, are cascadeable for improved performance and, once the weights have been calculated, are efficient in terms of their computation load in real time use.

Figure 6 shows the frequency response of a typical 100 point FIR filter. Note the improved roll-off rate.

Of all the digital signal processing techniques in general use, Fourier analysis is one of the most powerful. In Fourier analysis the signal is transformed from the time domain (amplitude versus time) to the frequency domain (amplitude versus frequency). Once in the frequency domain a variety of simple filtering techniques allow

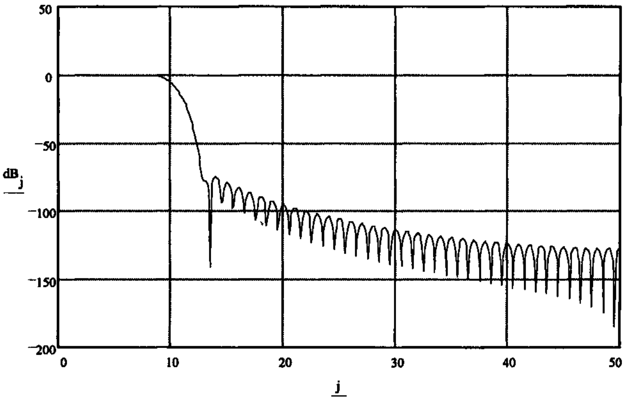

*Figure 6. Frequency response, 100 point FIR filter.*

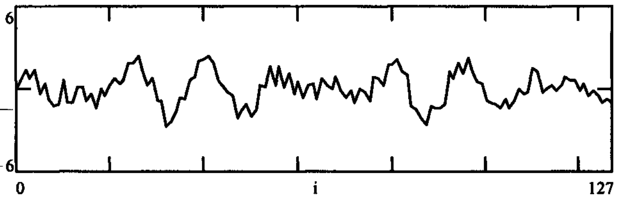

*Figure 7. Noise loaded signal in the time domain prior to Fourier analysis.*

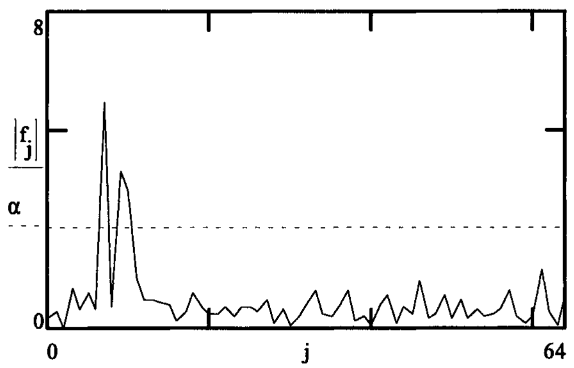

*Figure 8. Signal after transformation into the frequency domain. Note the threshold value α.*

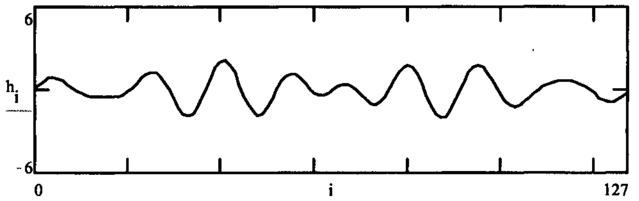

*Figure 9. Signal after shaping and inverse transform back to the time domain. Note the removal of higher frequency noise.*

the shaping of the required signal envelope. After the frequency domain data has been filtered the shaped information is then transformed back to the time domain. Figures 7, 8 and 9 show a signal prior to transformation, after its transformation into the frequency domain and after the inverse transform back to the time domain.

In this example the filter function is a simple application of the heavyside function to isolate the signal frequencies required, i.e.

$$a_j = f_j \Phi(f_j - \alpha)$$

The heavyside function acts to select frequencies in the frequency domain based on the amplitude of the signal compared to the threshold level $\alpha$.

Fourier transform is by its nature one of the more powerful signal processing techniques available. The down side is that the technique is *very* computationally intensive, with a typical 10 min chromatogram calling for in the order of $5 \times 10^8$ complex, i.e. real and imaginary, double-precision floating point multiplications. A secondary disadvantage is that because the technique requires the full data set (or at the minimum a large data set) and because of the computational intensity real-time operation is effectively impossible with current processor technology.

The authors do not propose to spend any more time enumerating all of the various digital signal processing methods, or their advantages or limitations. Reference to almost any digital signal processing text book will indicate the scope available. It is interesting to note, however, the practical results from digital signal processing. Figures 10 and 11 show a GC-FID chromatogram before and after digital signal processing.

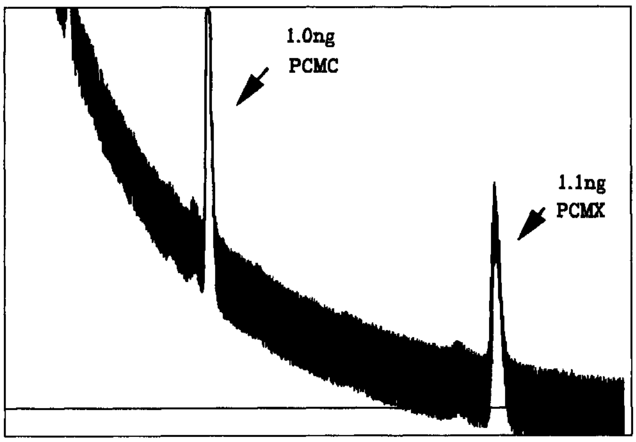

*Figure 10. GC-FID chromatogram prior to signal processing. Note the high level of noise.*

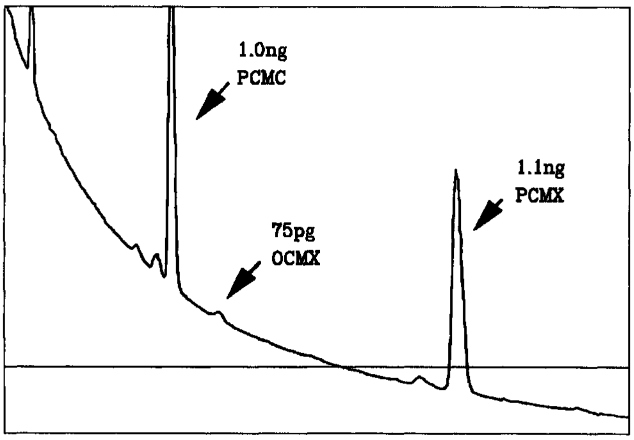

*Figure 11. The chromatogram after signal processing. With the noise stripped away the presence of a previously undetected compound is revealed.*

Thomas Swan have been using digital signal processing techniques for a number of years. With each new generation of systems the sensitivity obtained has been extended.

An added benefit of the signal processing approach is that the systems are 'insertive', i.e. the additional processing is inserted in the normal signal processing chain. This means that a single signal processor unit can be used with any chromatography system that can benefit from it rather than each chromatography system having to have its own dedicated signal processor. This helps offset the additional cost of the signal processing units and their associated PCs.

Early systems (many still in daily use) used 16 bit resolution digitized data with manual control of the signal gain and offset functions to allow us to provide the semblance of a higher resolution system. 386 and 486 based PCs were used to run the signal processing algorithms. These systems typically gave a factor of five-to-ten improvement in detection sensitivity on any given system. One major problem experienced with these early systems was the difficulty in handling the wide dynamic range produced by baseline drift due to both the chemical processes and the electronic drift in the detector amplifiers. Multi-component solvent gradient systems were especially difficult to work with!

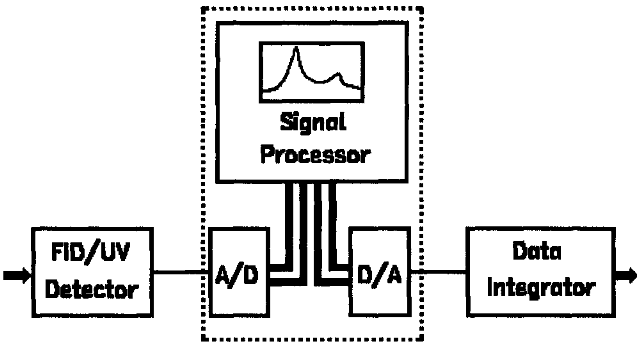

*Figure 12. 'Insertive' architecture used in the Thomas Swan signal process.*

Figure 12 shows the 'insertive architecture' used in the Thomas Swan signal processors.

The company's latest design is a multi-processor unit based around digital signal processor chips with a capability of up to 40 million 'real' floating point operations per second (40 MFlops) in the signal processing environment. The 'real' aspect of this performance is highlighted, as in a system environment it is surprising how many 'MIPs' and 'MFlops' can disappear into a normal PC system architecture. This new system, the ID/10, is designed to provide a computing engine to meet the demands of the signal processing algorithms that are operated now and those that the company might develop over the next few years. Allied to the DSP processor, an analogue input system with a 24 bit resolution and a 10 msec conversion time has been developed. With regard to noise, this new A/D system is remarkably clean with better than 22 bits free of peak-to-peak noise at a nominal 100 samples per second. A typical chromatography integrator based on current state-of-the-art semiconductor I/C technology may claim 24 bit resolution with 20 bits of noise free data, but this is normally based on 'RMS' measured noise. RMS measurements are fine for hi-fi but in chromatography where the signal shape is important, noise peaks are what show up on the chromatogram. Converting RMS noise to peak-to-peak noise (multiply RMS noise by 6·5) indicates that the typical integrator's claimed 20 bit performance is really closer to 16 bits where every lost bit of useable resolution effectively halves the potential sensitivity.

The ID/10 system also has a new suite of software adapted specifically to the DSP chip architecture. The software architecture uses a multi-stage frequency profiled filter approach with selectable user sample rate and 'enhance' factors. Figure 13 shows the frequency response of a typical stage in the filtering sequence. Note the rapid roll-off and high level of noise rejection both available and required to process 24 bit resolution data. Future algorithms are expected to include artificial intelligence techniques as well as a complex self-adaptive filtering approach.

An immediate benefit of the ID/10 is that the greater dynamic range allows the company to work directly with

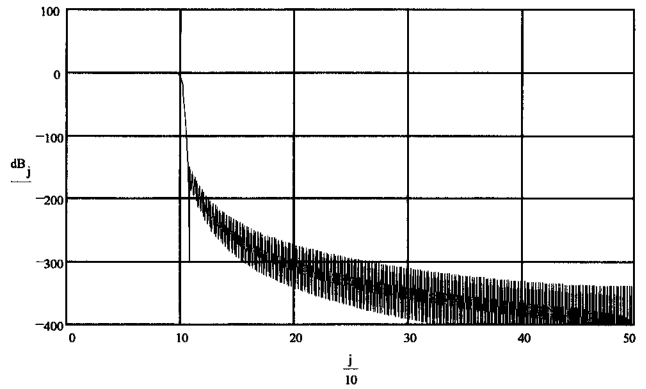

*Figure 13. Frequency response of a typical stage in the ID/10s filtering sequence.*

gradient systems and other systems with appreciable baseline drift without any loss of bottom end sensitivity.

A prototype ID/10 system has been used to process data from GC and HPLC systems. The following case studies demonstrate the improvement in sensitivity.

### Case studies

### *Liquid chromatography*

This case study investigates a mixture of four Phenols (Phenol, o-Cresol, m-Cresol and 3-Chlorophenol) in a water solution, using a liquid chromatography method. To aid measurement an internal standard (4-Chloro-3-Methyl Phenol) is also added to the solution. The system configuration consists of an ACS-352 ternary gradient pump system feeding a 50/50 Acetonitrile/water eluent at 1·5 ml/min to a C18 reverse phase column. The samples (20 µl) were directly injected onto the column. Detection is via a Thermo Separation Products Spectra 100 UV detector set for 280 nm with a sensitivity of 0·0005 AU full scale. Data integration is via a Spectra Physics Integration package using peak height as the measurement parameter. The study consisted of a series of experiments to find the limits of the system both prior to and after signal processing using a prototype version of the ID/10. From this study two cases were selected to show the level of improved sensitivity expected from signal processing. Figures 14 and 15 show chromatograms resulting from a run close to the limit of detection without signal processing. Peaks 1 to 4 (1 = Phenol, 2 = o-Cresol, 3 = m-Cresol, 4 = 3-Chlorophenol) reflect sample concentrations of circa 35 ppb. Peak 5, the internal standard 4-Chloro-3-Methyl Phenol reflects a concentration of 205 ppb.

Prior to signal processing, the chromatogram reflects a high level of noise. On repeat runs, this noise limits the repeatability of the measured peak height to approximately ±25%, indicating that this is the minimum level of detection for the system without signal processing. After signal processing, the repeatability of peak height measurement improves to ±5%. The chromatograms also show the level of noise reduction achieved by the signal processing.

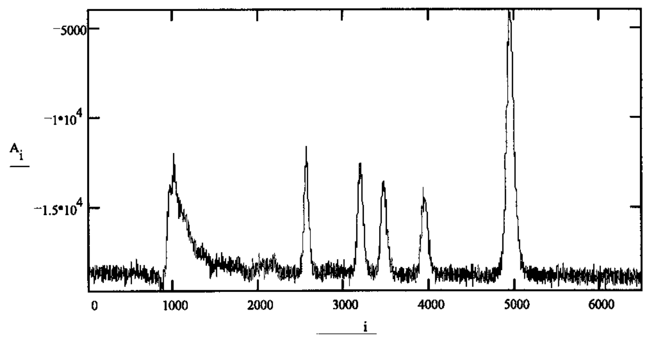

*Figure 14. A 35 ppb concentration phenols mixture HPLC chromatogram prior to signal processing.*

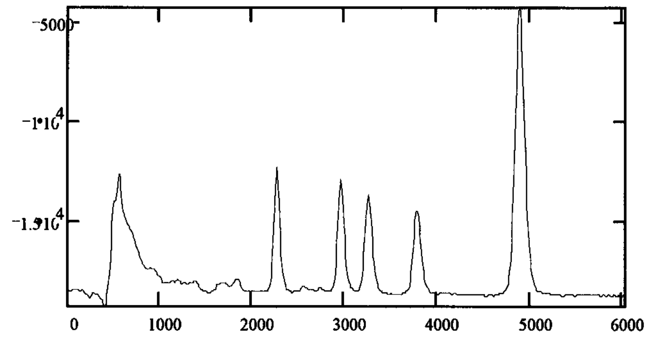

*Figure 15. A 35 ppb phenols mixture HPLC chromatogram after signal processing.*

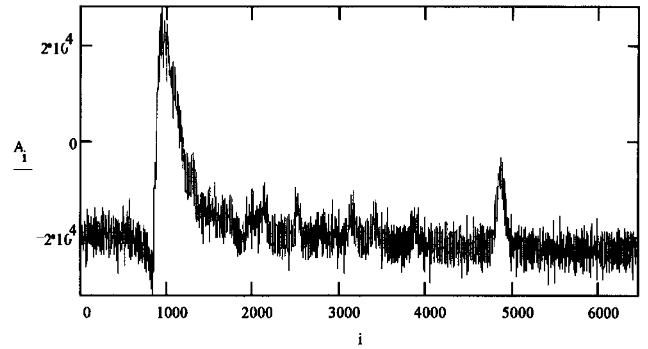

*Figure 16. A 35 ppb concentration phenols mixture HPLC chromatogram prior to signal processing.*

Figures 16 and 17 show the chromatograms resulting from a run closer to the limit of detection with signal processing. Peaks 1 to 4 reflect sample concentrations of *c.* 3·5 ppb. Peak 5, a concentration of 20 ppb.

Prior to signal processing, the chromatogram reflects a very high level of noise, indeed the sample is virtually unmeasurable. After signal processing, the sample is again measurable with a repeatability of peak height measurement of ±15%. Again, the chromatogram shows the level of noise reduction achieved. Also note the appearance of a series of additional minor peaks specifi-

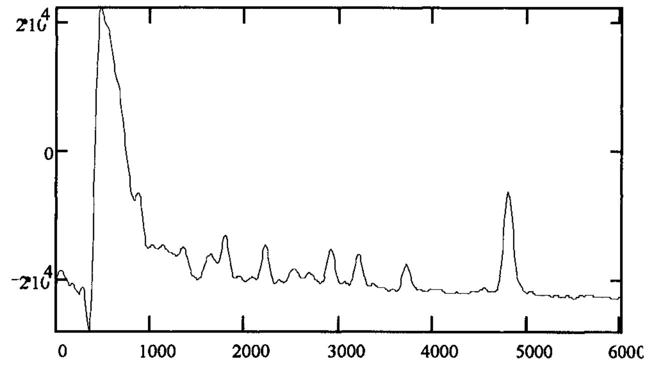

*Figure 17. 3·5 ppb concentration phenols mixture HPLC chromatogram after signal processing.*

cally in front of peak 1 and between peaks 1 and 2. These are attributable to impurities in the solvents used.

*Gas chromatographs*

This case study also investigates the mixture of four phenols (Phenol, o-Cresol, m-Cresol, and 3-Chlorophenol) in a water solution but this time using a gas chromatograph. Again to aid measurement the internal standard (4-Chloro-3-Methyl Phenol) is added to the solution. The system configuration consists of a Philips PU4500-FID chromatograph system (heavily modified) using hydrogen as the carrier gas (linear gas velocity 20 cm/s). The column used was a Econocap SE54, 0·53 mm I/D × 15 m long with a 1·2 μm film thickness. The analysis used a 0·5 μl sample via a splitless injection system. The temperature profile used was a 85–150°C ramp running at 8°C/min. Data integration is again via the Spectra Physics Integration package using peak height as the measurement parameter. Again the study consisted of a series of experiments to find the limits of the system both prior to and after signal processing using a prototype version of the ID/10. Figures 18 and 19 show chromatograms resulting from a run close to the limit of detection without signal processing. Peaks 1 to 4 (1 = Phenol, 2 = o-Cresol, 3 = m-Cresol, 4 = 3-Chlorophenol) reflect sample concentrations of c. 3·5 ppm. Peak 5, the internal standard, a concentration of 20 ppm.

Prior to signal processing, the chromatogram reflects a high level of noise sufficient to make the fourth peak (3-Chlorophenol) almost unmeasurable. On repeat runs, the noise limits the repeatability of the peak height measurement to approximately ±30%. After signal processing, the repeatability of peak height measurement improves to ±10% with the fourth peak becoming easily measurable.

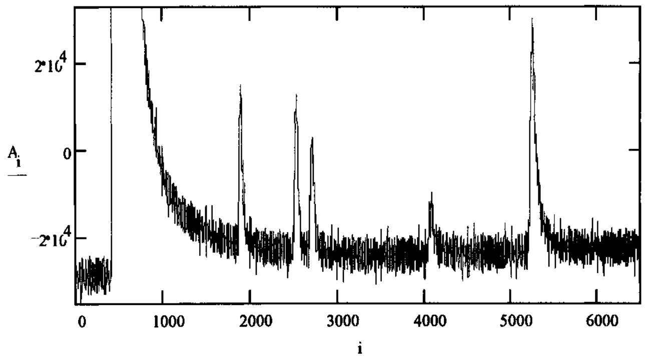

*Figure 18. 3·5 ppm phenols mixture GC-FID chromatogram prior to signal processing.*

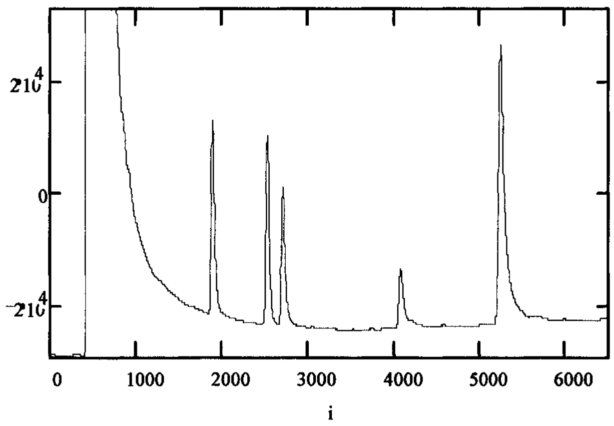

*Figure 19. 3·5 ppm phenols mixture GC-FID chromatograph after signal processing.*

Figures 20 and 21 show chromatograms close to the limit of detection with signal processing. In this case the Phenol, o-Cresol, m-Cresol, and 3-Chlorophenol sample concentrations were about 350 ppb and the 4-Chloro-3-Methyl Phenol 2 ppm. Again prior to signal processing, the signal is completely undetectable. After signal processing, the first three peaks are detectable with indications of the fourth peak, the 3-Chlorophenol. This chromatogram probably indicates the effective limit of the splitless injection technique for this sample rather than the limits of gas chromatography itself.

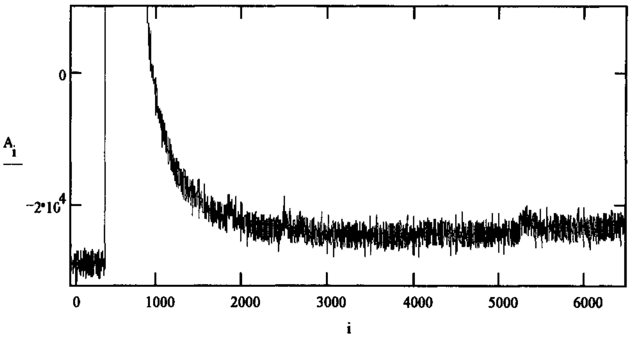

*Figure 20. A 350 ppb concentration phenols mixture GC-FID chromatogram prior to signal processing.*

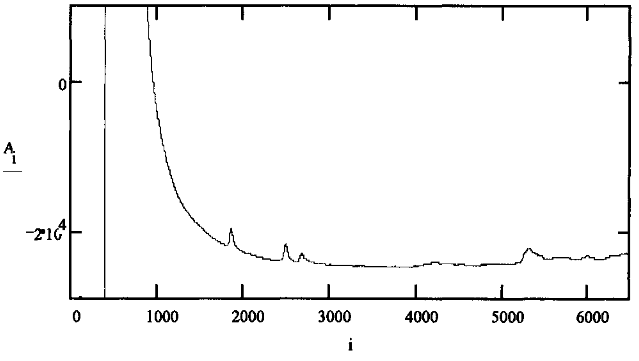

*Figure 21. A 350 ppb phenols mixture GC-FID chromatogram after signal processing.*

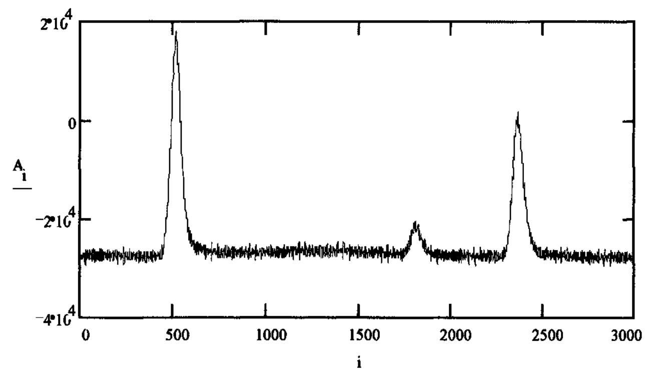

*Figure 22. VOC GC-FID chromatogram prior to signal processing.*

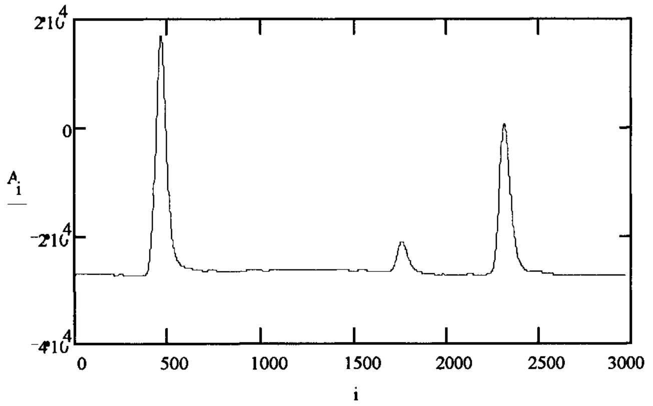

*Figure 23. VOC GC-FID chromatogram after signal processing.*

*VOC mixture*

This case study investigates the production monitoring of a volatile organic compound mixture containing toluene and perchloroethylene. In addition to the two expected compounds, the sample also included a third, so far, unidentified contaminant. The system configuration again consists of the Philips PU4500-FID chromatography system using hydrogen as the carrier gas (linear gas velocity 20 cm/s). The column used was a J&W DB5 of 0·53 mm I/D × 15 m long with a 1·5 µm film thickness. The samples (200 µl) were introduced without any prior preparation using splitless injection. The system was run isothermally at 65°C. Data integration was again via the Spectra Physics Integration package using peak height. Again the study consisted of a series of experiments to find the limits of the system both prior to and after signal processing. Figures 22 and 23 show chromatograms resulting from a run close to the limit of detection without signal processing. Peak 1 (Toluene) reflects a concentration of 10 ppb. Peak 2 (Perchloroethylene) a concentration of 100 ppb.

Prior to signal processing, the chromatogram again reflects a high level of noise. On repeat runs noise limits the repeatability of the peak height measurements to approximately ±15%. After signal processing, the repeatability of peak height measurement improves to ±5% with both peaks being easily measurable.

Figures 24 and 25 show chromatograms close to the limit of detection with signal processing. In this case Peak 1 (Toluene) represents a sample concentrations of circa

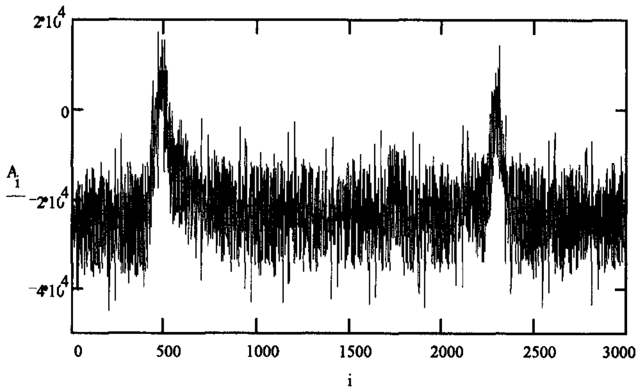

*Figure 24. The 1 ppb toluene VOC GC-FID chromatogram prior to signal processing.*

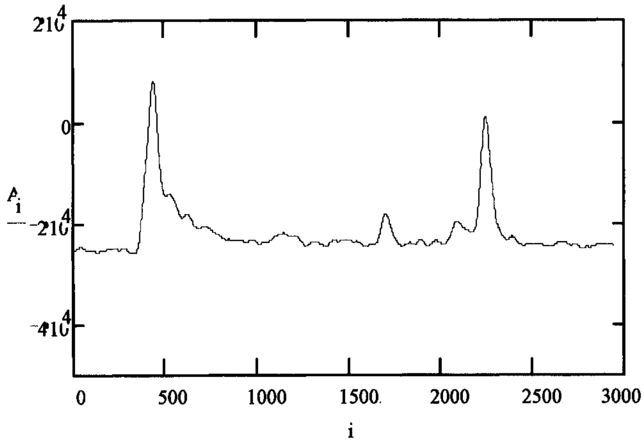

*Figure 25. The 1 ppb toluene VOC GC-FID chromatogram after signal processing.*

1 ppb and Peak 2 (Perchloroethylene) a concentration of 10 ppb.

Again prior to signal processing, the signal is virtually unmeasurable. After signal processing, the peaks are easily detectable with a repeatability of about 15%, although very low level impurities and other contaminants are starting to appear. This study indicates the strong feasibility of a production monitoring system using direct injection without any prior sample preparation.

### Conclusion

By investing in continual improvement and development of analytical equipment, a task more commonly left to the equipment's vendors, Thomas Swan & Co. have been able to reap many benefits. Some of these are simple, such as having the in-house skills and resources to maintain and repair equipment with the associated reduction in cost and downtime, or of being able to operate at state-of-the-art levels with lower cost equipment. Others are more complex, such as the benefit of extended equipment operating life or the reduction in the disruption costs associated with new equipment purchases. Overall, the greatest benefit, however, is that the required levels of sensitivity have been achieved without the day-to-day penalties of extensive sample preparation or exotic analytical processes.

